
JOURNAL INFORMATION
==============================
NLM Title Abbreviation: Med Chir Rev
NLM Journal ID: 0034617

The Medico-Chirurgical Review

Publisher: Biomedical Journal Digitization Project

ARTICLE INFORMATION
==============================
PMCID: PMC4966253
PMID: 29917624
Article version: 1
Subjects: Articles

Hospital Practice

Print publication date: 1828 Oct 1
Volume: 9
Issue: 18
First page: 471
Last page: 480
1828 Jul
License: This work is free of known copyright restrictions. For more information, please see PMC Back Issue Digitization.
License URL: https://creativecommons.org/publicdomain/mark/1.0/

==============================

medical remuneration, by which he is surrounded. But if he trusts to dress, address, whist-parties, and scandal-bearing, he may loudly declaim against the laws, whilst the fault is nearer home!

We confidently predict (and we do not speak from light authority) that the system of medical apprenticeship will be done away in an early part of the next session of parliament. This will be one of the most important measures for liberalising and improving the medical profession, that has ever been put in execution. This apprenticeship is not merely a waste of five years, but it is the foundation of an illiterate and unscientific disposition through life. It is almost impossible that the education of the general practitioner can be complete, or his station in society so good as it ought to be, while this mark of mechanical degradation is indented on parchment. No man should *bind* his son, for five years, behind a counter, who does not intend him to remain there as a chemist, all his life. As the Apothecaries' Company will, even now, admit five years' study, as an equivalent to five years' apprenticeship, (a great condescension certainly!) that man is mad, who would place his son under indenture, except on the express stipulation that he is to be free, after a year or two, to repair to the proper marts for medical science and literature.

6. *Strictura Orificii Urethræ.* Newton discovered the law of gravitation by the fall of an apple—Mr. Earle, the cause of a number of troublesome complaints, by being "disturbed in the act of making water." This cause is a natural contraction, either immediately at the orifice of the urethra, or a very little way from the orifice, behind which contraction, the urethra is of its natural diameter. The effects of this contraction are very deleterious, producing all the symptoms of the worst strictures, and *strictures themselves.* Irritable bladder, disease of the prostate, chronic affection of the testicles (sclerocele, or hydrocele) and great constitutional derangement, are the common consequences of this natural contraction of the orificium urethræ. These consequences are thought to be produced by the distention occasioned in the urethra from the check given to the current near the end of the passage. Mr. Earle thinks it induces far more serious disease in the urinary organs, and their appendages than stricture at any other part of the urethra. The remedy is simple —a division of the contraction, by means of an instrument which this ingenious surgeon has invented, and with which he operates in Bartholomew's Hospital.

### HOSPITAL PRACTICE.

32

### FOUNDLING HOSPITAL. M. BARON, PHYSICIAN.

1. *Gastro-Enteritis of Infants.* Mr. Savatier reports from the above hospital on the subject of gastric and intestinal inflammation among newly born children. The following case is introduced from one of the wards of M. Baron, as an example of what the reporter denominates: "the first (slightest) degree of gastro-enteritis," in this class of subjects. On the 21st of April, was seen in the above ward, a child about six weeks old, presenting the following symptoms:—tension, and general, though moderate heat of the abdomen—intense redness of the anus and vicinity—tongue red at the sides and tip, white at the back and middle part—diarrhœa of greenish matters—constant, and almost tetanic rigidity of the muscles about the back of the neck —countenance expressive of suffering. *Abstinence—fomentations to the abdomen —syrup of mallows with gum.* In ten days, the symptoms were all dispersed. Each day the tongue was observed to be less red, the belly less tense, the rigidity of the muscles above-mentioned less tetanic.

In the winter of 1824-5, M. Savatier states, that he attended a great number of children affected with convulsions. It was observed, that these children first shewed symptoms of disinclination for the breast; were very fretful; had the tongue red at the sides; the belly tense and hot; the anus red, with moderate diarrhœa. Leeches, except in a very few cases, were not prescribed, on account of the prejudices against them. Fomentations, tepid baths, and diluents effected the cure, with abstinence from the breast.

We are inclined to agree with the reporter, who declaims against the "MEDICINA PERTURBATRIX" in such tender subjects. It is very true, that in such subjects, diseases make rapid progress—but it is equally true that they are easily counteracted by gentle means, and rapidly exasperated by irritating medicines, especially purgatives.

The reporter observes, that there is a grade of gastro-enteritis in the newly-born, which is, perhaps, still *lighter* than the foregoing. M. Baron is accustomed to examine foundlings before they are sent off into the country, and often finds them presenting redness of the sides and tip of the tongue, with similar redness of the anus. When to these signs is added any tension of the abdomen, or diarrhœa, Dr. B. considers that there is inflammation of the mucous membrane of the stomach and bowels, and retains them for medical treatment. The reporter properly observes, that medical men should be in the habit of inspecting the tongues of children that are in health, otherwise they will not be able to properly appreciate the appearances in those who are ill. In children, unless the gastro-enteritis be very intense, it is rare to find the tongue dry. The state of the mucous membrane of the lips will often prove a guide in our diagnosis. M. Savatier thinks that the above slight grade of gastro-enteritis is caused by the sudden change from the INTRA to the EXTRA-UTERINE life. The action of the air itself, especially if cold, may induce this excitation of the mucous membranes, through sympathy with the skin, which is often but badly defended against aerial vicissitudes. Even the entrance of cold into the lungs, where the surface is sufficiently defended by cloathing, may prove the cause of inflammation of the mucous membrane of the bowels. In most cases, a few days sojourn at the Hospice, enables the physician to discharge the infants to the country to be nursed. In others, the phlegmasia makes progress, and the infant is taken to the infirmary of the Hospice, with the symptoms described in the above case, except the rigidity of the muscles.

The *second degree* of intensity of this affection among new-born children, is exemplified by the following case. A female child, eleven days old, was brought into the infirmary of the Hospice, on the 19th March, 1827, presenting the following symptoms :—diarrhœal discharge of yellow and watery matters—tongue red at the edges, and white on the surface—abdomen not apparently tense—heat of skin natural—little or no crying—pulse accelerated—thorax sounding well in all points. *Rice-water with gum—abstinence.* 20*th* and 21*st.* Continuance of diarrhœa—drink vomited—the abdomen somewhat hotter than the other parts of the body. *Fomentations to the abdomen.* 22nd. Pulse still more accelerated. Death, without any convulsions.

*Dissection.* The child was very robust in appearance. The lungs were extremely gorged with blood, and not crepitous in any part, but without mark of inflammation. The heart and large arteries were also much gorged, as were the veins. The mucous membrane of the stomach was of one uniform redness throughout, with arborisation of the vessels—mesenteric glands enlarged and inflamed—nothing particular in the small or large intestines, liver, or spleen.

The above case, the reporter observes, presents an exact representation of the symptoms and post-mortem appearances, in the second degree of infantile gastro-enteritic inflammation.

*Third degree*—or *superacute form.* Infants are frequently brought into the infirmary, who have been put to bed in the ward of the CRECHE the preceding night, apparently well; but who, nevertheless, are found dead the next morning. The reporter has examined many of these, and has invariably found the vessels of the gastro-intestinal mucous membrane extremely injected—often from the œsophagus to the rectum; but most commonly confined to the stomach, and the lower portion of colon. The redness is sometimes confined to the mucous membrane, but occasionally penetrates through the muscular and peritoneal tunics, which are of all shades, from a brown to a black hue. To this species of gastro-intestinal apoplexy is generally joined a very acute degree of carditis, characterised by high vascular injection of the reflected portion of pericardium, and the coats of the great vessels, with effusion of serum in abundance. Still-born children have often presented these appearances. He was doubtful, at first, whether these phenomena might not be caused, or take place in the agonies of death; but attentive observation and reflection convince him, that they are the result of inflammation.—Clinique.

In closing this short, but interesting notice, from the wards of M. Baron, we may remark, that tubercles are not unfrequently found in the lungs of the new-born children brought to the Hospice—proving that tubercular phthisis (or, at least, the seeds of that disease) may be congenital.

How is it that we have few or no reports—few or no investigations, from the wards of our numerous Lying-in charities in this metropolis? Do the medical officers keep the knowledge acquired by dissection in their own breasts, for the sake of raising themselves above their neighbours in pathological science—or are they too lazy, too busy, or too *sleepy*, after their *labours*, to plod in the dissecting room? None of these! Their zeal is generously and humanely directed to the *cure* of all these gastro-enterites of the new-born, by a course of active and polypharmic remedies, which would astonish M. Le Docteur Baron, and his disciple Savatier! Possibly these dissections of our Parisian brethren may open the eyes of London practitioners to the danger of pouring medicines on stomachs, such as we have here described.

---

### 33. Fistula of the Parotid Duct and Gland.

[La Pitié.]

M. Mirault has published an extensive memoir on the process employed by M. Deguise, as modified by Beclard. The following case will exemplify the process in question.

*Case.* Auguste Gonoreau, aged 18, the clerk of a notary, lacerated his cheek by falling upon an iron stove. One of the upper screws penetrated the cheek, wounding the buccinator near the masseter, though it does not appear that it reached the mouth. The wound healed rapidly, with the exception of a small fistula through which there was a discharge of a clear liquid, especially while he was eating. This showed that the stenonian duct had been wounded. After having fruitlessly employed the several processes which were directed by his attending surgeon, on the 21st of May, 1821, he placed himself under the charge of Beclard, in *l'Hôpital de la Pitié*. It had been three years since he had received the injury.

Beclard converted the fistula into a recent wound, by the excision of its sides; then with a small trochar he perforated the cheek from the wound, passing the instrument obliquely backwards. Having withdrawn the stilette through the canula which remained in the perforation, he passed the end of a leaden wire. Having withdrawn the canula, he in the same manner made a second puncture; beginning, however, within the mouth, about three lines anterior to the former puncture, carrying the point of the instrument obliquely, till it passed into the wound near the place where the wire was inserted. The external end of the wire was passed through this opening into the mouth, being thus bent somewhat in the form of the letter V. The ends of the wire were twisted together; and the external wound was closed by a needle and the twisted suture. For the first three days, there was some distention and pain, owing to the saliva's not passing at first very readily by the side of the leaden wire. On the fifth day, the saliva passed freely into the mouth, and the swelling was nearly gone; the external wound had united, and the needle was now removed. Still, the internal fistula was not so complete as to carry off all the saliva, while the patient was eating. The duct was distended into a kind of sac, producing, during his meals, a small tumour, which was, however, easily emptied into the mouth, by a very slight external pressure. The leaden wire remained inside the cheek, till it fell out, in September, when the cure was complete.—*Archives Generales.*

---

### 34. Phlebitis.

[Hopital de la Charité.]

There is certainly something peculiar in inflammation of the venous tubes, whilst the generally fatal nature of the malady makes it one of considerable interest both to surgeon and physician.

It appears that pregnant women, or rather, women *after* parturition, are liable to inflammation of the veins of the uterus, the pelvis, or the thigh. The cases which have been lately brought forward by Dr. Davis, and some of his supporters, whilst they failed in establishing the Doctor's theory of the *phlebitic* origin of phlegmatia dolens, demonstrate in a conclusive manner, how frequent and how fatal an affection is inflammation of the femoral and iliac veins. A very similar case occurred to M. Fouquier, at the hospital of La Charité, and is reported in a recent number of the Clinique.

*Case* 1. A woman, about thirty years of age, was brought into the hospital on the tenth day of her confinement in a condition approaching to stupor, and incapable of making correct replies to the questions that were put to her. The pulse was rapid, the surface parched and of a cadaveric hue, the tongue coated with mucus, and rather dry. There was pain in the abdomen on pressure, and a circumscribed, round-shaped tumour in the pelvic basin; both the lower limbs were œdematous, and in the position of semi-flexion, any attempts at extending them occasioning acute pain. The respiration was natural, and the patient appeared to suffer little.

The pain in the abdomen and tumour in the pelvis led to the suspicion of peritonitis, complicated with inflammation of the uterus. Leeches to the abdomen, emollient drinks, and broths were ordered, but the symptoms became worse, the tongue was dry and brown—the teeth incrusted—the pulse innumerably rapid—the senses wandered—the patient lay continually on the side or belly, and on the 15th day after her admission she expired.

*Dissection four and twenty hours after death.* Traces of arachnitis were detected in the head, but nothing particular in the substance of the brain. A trifling quantity of bloody serum was discovered in the pleuræ; the lungs were not so crepitating as they should be, and on cutting into them a considerable quantity of yellow fluid, mixed with streaks of blood, flowed out. This engorgement of the lung was most perceptible on its posterior aspect. The mucous membrane of the stomach and intestines was unaffected, with the exception of some reddish patches of small extent upon the colon. The peritoneum was not at all inflamed, but the uterus was double its natural volume, pale in colour, soft in texture, and its inner surface coated with a layer of greyish pulpy matter. The vessels, though distinct, were empty, and the uterine and spermatic veins, being opened in their whole length, presented not a trace of inflammation.

The femoral vein and its divisions on either side were thickened, red on their internal coat, and filled with clots, which, in the upper part of the tubes especially, had purulent matter in their centre. The iliac veins were also reddened, coated within by a membranous layer of lymph, which extended to the inferior cava, and filled with a grey-coloured, bouillie-like fluid, which, above, was evidently sanious, and diluted with the blood of the vena cava.

The reporter remarks upon the case, and we entirely agree with him, that phlebitis does not prove fatal, merely as an inflammation, but from its introducing a quantity of pus into the current of the circulation, to mix with and *poison* the constitution of the blood. Whoever has seen many cases of phlebitis, must be struck with the rapidity with which depression supervenes. The pulse, which, for a day or two, often not so long, was full and bounding, changes to a rapid, wiry, and feeble beat; the surface of the body at first grows pale and pinched, and latterly assumes a decided bilious tint; the pain, which, in the commencement was frequently severe, becomes either masked or lulled, the senses wander, and the patient dies delirious.

It is a curious fact, but one which is well established, that abscesses and deposits of lymph in the structure of the lungs, which are not uncommon after surgical operations, almost always occur in a greater or less degree, in cases of phlebitis. How is the fact to be explained? Are we to imagine that a certain undefinable sympathy exists between the condition of the vein and the pulmonary apparatus, or adopt the humoral pathology, and conclude that it depends on the vitiation of the fluid? We cannot help inclining towards the latter, for surely if the venous blood is altered by an admixture of pus and lymph, it cannot be in a proper condition to be carried into the delicate structure of the lungs, nor is it likely to prove harmless to that structure in its passage. Another circumstance that would seem to be in favour of the humoral doctrine is the fluidity of the blood after death, which is always remarked in phlebitic patients, and of which we detailed a very decided example in the 17th number of the Journal.

Granting, however, that it is the vitiation of the fluids which so rapidly prostrates the powers of life, still this vitiation is a secondary process, and the formation of pus and lymph are only the results of preceding inflammation. This being the case, it appears, that if more powerful depletion, and especially locally, were employed at the onset of the disease, we might have a more favourable prospect of arresting it in limine, and preventing the purulent effusion. Had we a case of inflammation of the saphena, or veins in the upper extremity, we should cover the limb with leeches, not as they are commonly employed, by twos or threes, a mere apology for blood-letting, but by forties! We should also be inclined to give a trial to the application of cold, especially above the part immediately inflamed, and, if the state of the limb admitted it, apply compression, in order to promote the deposition of coagulum, and formation of adhesion between the inflammation and the heart. General blood-letting should be cautiously employed, as the patient but seldom bears it well when the inflammation has gone beyond its earliest stages.

It is singular that inflammation of the femoral and iliac veins should be so frequent a consequence of child-birth; but this, at least, is evident from the case at the head of the report; that the affection of the vessels in question may occur without the slightest appearance of phlegmatia dolens. In the case it is merely observed, that the lower limbs were *œdematous*, and painful when extended, though the very essence of Dr. Davis's pathology of phlegmatia dolens, viz. inflammation of the crural veins, was there

In a case recorded from St. George's Hospital, in the Medical Gazette, a short while ago, there was inflammation of the crural vein as high as the profunda, and yet the limb was scarcely swollen, and had not the slightest character in common with phlegmatia dolens. We have always opposed the theory, and we doubt whether the Doctor himself would venture to maintain it now. It is laid in the tomb of all the Capulets, and we shall be loth again to rake up the ashes of the dead!

*Case* 2. A country girl, affected with white swelling in the knee-joint, suffered amputation of the thigh, on the 19th of May, at the Hospice de Perfectionnement. Two arteries only were tied at first, but at the end of an hour and a half, the dressing was completed and additional ligatures applied. The patient bore the operation badly, and was affected in the evening with flushing of the face, quick pulse, and vomiting. Blood was abstracted from the arm, but she passed a wretched night, suffering from cephalalgia, vomiting, and pain in the stump.

On the 20th, she was worse; the tongue was dry and red, the thirst ardent, the vomiting incessant, the face was flushed, the surface of the body hot and parched. The dressings on the stump were stained with blood, and arterial looking blood made its way between the bandages. The bleeding from the arm was repeated, and leeches applied to the head, but without relief. Depression, both physical and moral, supervened—the skin grew pale—the countenance sunken and expressive of extreme anxiety—the tongue was dry and brown—the bowels obstinately costive. The pain in the stump was always great, and its surface white and sloughy. On the 26th delirium was established, and on the 27th, eight days after the performance of the operation, the patient sank.

*Dissection.* The vessels of the pia mater were rather injected, and the cerebrum, when cut into, was studded with bloody spots. The heart and lungs were sound, but the pleura on the left side of the chest was covered with recent adhesions. In the neighbourhood of the valve of the colon follicular ulcerations were observed, apparently advancing to cicatrization, but the rest of the abdominal viscera were sound.

On examining the stump, the periosteum was found to be loosened from the bone as high as the lesser trochanter. The femoral artery was filled at its extremity by a vermiform clot which adhered to the sides of the vessels, and extended into the cavity of the nearest collateral branch. Three inches of the femoral vein were filled with pus, but its tunics were unaltered in consistence. At the point which was in contact with the pus, the internal coat was paler than it should be, though above it was injected and the colour of the blood which it contained. This staining and discolouration extended to the iliac vein, and even to the cava.

We cannot but conclude, from the identity of symptoms with those of phlebitis, that the above was a case of inflammation of the femoral vein. It is singular that the coats of the vessel, which for full three inches was filled with pus, should have been so unaltered as they were.

In the cases related above the inflammation was seated in the lower extremities, but in those which we are going to detail it followed venesection in the arm.

*Case* 3. A young woman, ætatis 22, of good constitution, and in the fifth month of utero-gestation, was bled in the arm at the Hospice de Perfectionnement, for oppression and pain in the left side of the thorax. Two days afterwards she returned with fever, swelling of the arm, especially around the puncture, and discolouration of the skin in the situation of the veins. Leeches were ordered, but not applied, and on the next day (the 14th Jan.) the swelling had increased. The limb was rolled from the finger to above the affected part, but the symptoms still persisted, and the pulse was 110 in the minute; poultices were applied, and on the 16th, five and twenty leeches placed upon the arm. The pain, &c. in the arm was in part relieved, but the cough grew worse, the tongue was red and dry, the pulse 120, excessive agitation. On the 18th, the opening made by the lancet was enlarged, and issue given to a quantity of bloody pus, when the swelling of the arm diminished. She now complained of pain behind the sternum, and 20 leeches were applied. The cough, however, continued troublesome—she suffered from a burning thirst, and though the inflammation in the vein diminished, the pain in the sternum was unabated. *Twelve more leeches—blister to the chest.*

On the 25th, contractions of the uterus came on, and on the 26th, the poor young woman was in a dying state. The uterine contractions were still very powerful, and the neck of the uterus not dilating, M. J. Cloquet divided it to the extent of a quarter of an inch, when the contractions of the organ were redoubled, and after a little while the patient was delivered of a child, which lived three hours. About this time the mother too expired.

*Dissection.* All the veins from the arm to the heart were inflamed, and filled with a greyish pus; the right ventricle itself presented marks of inflammation, and was filled with clots of blood and pus, which were also found in the pulmonary artery. The left lung was inflamed, and a tubercle existed in the right.

Another case of phlebitis after venesection is related in the Medical Gazette.

*Case* 4. An athletic young man, a "traveller," was admitted into St. George's Hospital, on the 18th of June, with general anasarca and a dry cough, for which he was ordered to be bled by Dr. Hewett. The bleeding was repeated, and on both occasions the blood was abstracted from the same opening in the median cephalic of the right side. On the 21st, he complained of pain and tenderness in the arm, which was evidently swollen. The lips of the puncture were adherent, the integuments around a little red, the pulse 112, rather full and easily compressed, the tongue white, the bowels costive. Sixteen leeches and afterwards a poultice were placed upon the arm, and the bowels opened by scammony and blue pill. A draught of aq. ammon. acet.—potass. acet. ℈j. syrup. aurantii ℈j. was also ordered every six hours.

Next day, a red streak was noticed running from the puncture downwards to the wrist, in the line of the cephalic vein, which was painful upon pressure. The bowels were freely opened, but he was troubled with a vomiting of green and bilious looking fluids. *Twelve leeches to the arm—magnes. ust.* ℈j.—*pulv. tragac. c.* ʒ*ss.—liq. potas.* ♏ xij.—*syr. alth.* ʒij.—*aq. menth. vir.* ʒ x.—*t. opii* ♏ ij. *statim. et o. h. repet. quamdiu perstet vomitus.*

In the evening he was ordered calomel and opium, but on the 23d the arm wore a most alarming aspect. A sero-purulent discharge was continually oozing from the lancet-wound—the fore-arm was swollen and tender to the touch—the arm was also tender as high as the insertion of the deltoid muscle—and red lines coursed upwards and downwards in the direction of the cephalic, the median cephalic, and median veins. The pulse was 96, and there was cough excited on taking a full inspiration. Mr. Rose was requested to see the case, and immediately enlarged the puncture, which gave issue to a mixture of serum, pus, and blood. The coats of the vessel were greatly thickened. The pain and tenderness of the arm were much relieved by the opening which was made, but in the course of the afternoon some hæmorrhage occurred, which returned during the night in considerable quantity, and was stopped by pressure.

On the 24th, all the symptoms were exasperated—the pulse innumerably rapid —the pain had disappeared—the body bloated and of a bilious tint—the senses wandering—the countenance expressive of intense anxiety. At 6, P. M. he died.

*Dissection.* A pint of discoloured serum in the right side of the chest, and extensive, but not very recent, adhesions ; on the left side the same appearances in a less degree. The lower lobes of both the lungs, especially the right, were fleshy and consolidated, the mucous membrane of the bronchi and trachea was injected, the pericardium more filled with water than it should be. There was a general disposition to fluidity in the blood, and the lining of the left ventricle, as well as the inner coat of the thoracic and abdominal aorta, were stained of a cherry tint.

The median cephalic (in which the puncture had been made) was exceedingly thickened in its coats, and so was the cephalic trunk as high as the insertion of the deltoid. The inner coat of the cephalic was inflamed to within two inches and a half of its junction with the axillary, above which point no appearance of disease could be detected. Very little pus existed in the veins, but the cephalic trunk *below* the spot where the median cephalic joined it, was inflamed on its inner surface, and its cavity plugged up by coagulable lymph. No adhesion had been formed between the coats *above* the puncture, so that there remained a free and unobstructed channel for the escape of the blood from the axillary trunk. The median basilic was thickened and inflamed, and the basilica itself shewed feebler traces of inflammatory action. The cellular membrane around the brachial vessels was injected, though the vessels themselves were sound.

The reporter remarks upon the case, that " the thoracic inflammation, particularly on the right side, where it was most acute, we must suppose to be in part a sequence of the affection of the vein, as in the generality of cases of phlebitis after venesection, it has been found to have occurred. A patient was admitted into the hospital last year, who had swallowed oxalic acid, and he had nearly recovered from the effects of the poison, when he was ordered to be bled. Inflammation of the vein, (the median cephalic) supervened, and the patient died. On dissection, the cephalic vein was filled with pus and lymph, and marks of the most intense pleuritic inflammation were discovered in the side of the chest corresponding to the arm he had been bled in. In two of the cases recorded by Mr. Hodgson, the same appearance was observed ; and in the instance of phlebitis of the thigh, reported in our last, the pleuræ were inflamed and abscesses forming in the lungs."

We believe that there is truth in the observations of the reporter, but still it is curious that the tables may be turned upon them. It has been remarked, and by no less an authority than Sir Astley Cooper, that inflammation of the veins in the arm after venesection is much more frequent when the patient is bled *for* thoracic inflammation, than for any other disease whatever. Whether the condition of the lungs and vein act and re-act upon each other is a point which we leave where we find it.

---

### 35. Fracture of the Spine.

### [St. George's Hospital.]

An interesting case of the above very melancholy accident was admitted a little while ago, under the care of Mr. Brodie.

*Case.* Michael Williams, ætatis 71, had been working on a hay-stack at Acton throughout the day, when he fell asleep, and slipped from the stack upon the ground. He was taken up next morning, (26th of June,) and carried to the hospital, presenting the following symptoms.

No motion whatever in the limbs, excepting flexion and rotation of the left fore-arm. Sensation was destroyed in the lower extremities and in the trunk about as high as the 4th dorsal vertebra. It was lost too in the right arm below the elbow, and very imperfect above it; in the left arm there still remained some feeling, but it was considerably duller than it should be. The breathing was oppressed, and solely performed by the diaphragm, so that the ribs were depressed instead of elevated during inspiration. There was occasional priapism—surface rather cold—pulse small and feeble—senses perfectly collected.

On examining the spine no crepitus or displacement could be felt, but considerable pain was experienced on making pressure above the 5th dorsal vertebra. On introducing the catheter, about a pint of heathy urine was drawn off.

*Mist. ætheris, c. mist. camph.* āā ℥vj. *6tis horis.*

*Vespere.* The pulse is 80, and rather stronger, breathing more laborious, and consisting of twenty-six respirations in the minute, the tongue, which in the morning was brown and dry, is rather moist.

*27th*, A.M. The breathing is still more laboured—pupils contracted—bowels not yet open—pulse 80—tongue loaded, but moist. On drawing off the urine, it tinged the litmus paper red. He continued sensible, but gradually sank, and died at 6, P.M.

*Dissection.* There was fracture of the spinous process of the 5th dorsal vertebra, and 6th cervical, but the principal injury was inflicted on the latter. The intervertebral cartilage, between the 5th and 6th cervical vertebræ, was torn across, so that about half an inch of the theca was exposed, and the articulating processes and bodies of the vertebræ were so widely separate, that the finger could be placed betwixt them. The theca vertebralis we have stated was exposed, but it was entire and unaffected, with the exception of a little ecchymosis. The medulla itself appeared to have escaped, but on cutting across it, opposite the seat of injury, its centre was of a brownish red, as if a slight effusion had occurred from the minuter vessels.

Nothing particular was discovered in the brain, the thorax, or abdomen.

In a clinical lecture delivered on the case by Mr. Brodie, he remarked that symptoms of fracture of the spine may follow an extravasation of blood within the theca, which is gradually absorbed. A person, for instance, may become paraplegiac after an injury to the back, but in two or three weeks the paralysis is relieved, and he walks as well as ever.

In fractures of the vertebræ, the animal heat is frequently higher than the natural standard, the limbs appearing to be burning hot. In a case which occurred to Mr. Brodie, the thermometer placed between the scrotum and the thigh immediately rose to 111, and continued at that point, even *after* the patient's death. When the fracture is low in the cervical vertebræ, the pupils are generally contracted—the intellectual faculties, which were sound at first, become disordered, and the patient dies, in general, within the first three days, and often in twelve hours.

When the injury is lower in the back, the person may linger for a week or more, or even recover entirely, save that the limbs continue paralytic. An example of recovery occurred at the hospital last Summer, in the case of a little boy, who was admitted with fracture of the dorsal vertebræ, attended with evident displacement. Extension was employed, and the displacement diminished in a slight degree, but the boy was paralytic in his lower limbs. He remained in the hospital about four months, and, when he left it, his health was good, and the paralysis much relieved. Another instance of recovery occurred to a surgeon in the country.

A man was exposed to an injury of the spine; and, on examination, the practitioner discovered that the first of the lumbar vertebræ projected beyond the level of the last of the dorsal. The gentleman reduced the dislocation or displacement, whatever it might be, and a year after the accident, the patient was admitted into St. George's with paralysis of the lower extremities. He remained in the hospital a little time, and, when dismissed, the paralysis was considerably relieved.

With regard to the pathology of the accident, if the patient dies within the first five days, the centre of the spinal marrow, opposite the seat of injury, is of the consistence of cream, and of a brownish colour. If, however, he survive for a month or two, the softening has proceeded to a greater extent, and the medulla, at the affected part, will float in water, and look as if entirely dissolved. The membranes of the medulla are by no means so frequently the seat of inflammation as the membranes of the brain.

Mr. Brodie made several remarks on the secretion of the urine, which deserve attention. In some cases, the majority, indeed, the urine is extremely ammoniacal, and deposits a quantity of ropy mucus, which, according to Mr. Brodie, will happen alike, whether the injury is high or low, whether the catheter is regularly employed or not. In other cases, the urine has an opaque and yellow hue, and acts as an acid on the litmus paper, a condition which may alternate with the ammoniacal on different days. When the urine has been ammoniacal, the mucous membrane of the bladder is found to be inflamed, and its vessels filled with blood. When, on the other hand, the urine has been acid, the vessels are actually empty, and the mucous membrane of the bladder preternaturally yellow, and *dyed*, as it were, by the yellow urine. In some instances, no change whatever of the mucous membrane is discovered. The same appearances are noticed in the lining membrane of the kidneys, which are generally unnaturally soft, especially when the urine has been ammoniacal.

Mr. Brodie regretted that so little could be effected by the surgeon in the treatment of the injury. If there was displacement of the bones, he would reduce it, as the reduction was followed by relief in the cases of the man and the boy, which are alluded to above. Abstraction of blood, if the symptoms should indicate inflammatory action—the horizontal posture—purgation, when the bowels are confined, and the regular employment of the catheter, are all that Mr. Brodie can suggest. To trephining Mr. B. is decidedly opposed on the ground that he never saw a case where the operation could have possibly done good. If only the arch of the vertebra is driven in upon the marrow, we might suppose the trephine would be of service; such an accident, however, must be extremely rare, and, even if it did occur, it would be impossible for the surgeon to distinguish it from that in which the body of the vertebra is broken also.

Some other objections to the operation were urged by Mr. Brodie, but none were so strong as the above.

---

36. Simple Fracture of the Tibia, said to be followed by Delirium Tremens.

[Guy's Hospital.]

We know not how it is, but we are weekly cannonaded with cases of delirium tremens, as a consequence of injuries, in the *East*-end hospitals. Whether it be that the *gentlemen* received in those establishments are gifted by Nature, or endowed by gin and bitters with a strong predisposition to the affection in question; or whether, on the other hand, the gentlemen who report have a happy facility of seeing and discovering what their neighbours would not, is a point which may admit of disputation. For our own parts, we confess that we never saw an instance of delirium tremens, or any thing approaching to it, as an unequivocal consequence of injury, although we have been witnesses of dislocations and fractures in abundance! We have seen delirium after accidents, (and who has not?) but it was only a symptom of the general shock which the system had received, and we should no more have dreamed of considering or calling it delirium tremens, than we should have conceived the idea of christening it the chicken-pox. The delirium traumaticum of Baron Dupuytren is not delirium tremens, as it was by no means confined to individuals in the habit of drinking.

*Case.* A man, ætatis 42, who had for many years been given to drinking hard, and "was often in the habit of walking in the night in a state of delirium"(!!) was received into the hospital, on the 20th of June, under the care of Mr. Morgan, in consequence of a simple fracture of the tibia, situated "about three inches above the tibio-tarsal articulation," *Anglicé*, the ankle-joint. The tongue was furred—the pulse small and frequent—the aspect pallid—the eyes were "quick and bright"—the pupils much contracted. The fracture was reduced, the limb being put in splints, and placed upon the heel.

On the 21st he was hot, feverish, and so "furiously delirious," as to rise from bed and walk with the splints about his leg. There was tightness in the head—small quick pulse—furred tongue—contracted pupil. The countenance was flushed, and he was extremely tremulous, and constantly muttering to himself. He was ordered two ounces of julep of ammonia, with four drops of the liquor opii sedativus every four hours. The delirium continued, but diminished in intensity—the quickness of the pulse subsided, and the tongue grew clean. Twenty minims of the tincture of hops were added on the 26th, to every dose of the medicine before prescribed; and on the 30th, the delirium had left him, the febrile excitement was gone, and the leg was doing well.—*Lancet.*

The delirium which followed the accident (give it what name we may) was certainly peculiar in its characters. It would seem by the report that the patient was previously affected with it, at least he was "in the habit of walking in a state of delirium in the night," a circumstance which renders it very difficult to determine how much of the affection depended on the injury.

### 37. On Excision of the Vas Deferens as a Substitute for Castration.

It appears that Mr. Key has performed the above operation, in an instance of disease in the testicle, which had resisted every other kind of treatment. The effects of the operation are not detailed, and we venture to pronounce that it never will succeed. We cannot conceive, in the remotest degree, what the principle of the measure can be; nay, we will go so far as to say, that it is in absolute [illegible]nce of principle of all kind.

Would the stoppage of the ureter be likely to cure or to cause a disease of the kidney? The answer is easy enough. Look to what organ of the body we may, it invariably happens that disorder or disease of the secreting apparatus is produced, if the excretory duct is choked up or obliterated. The surface of the body, the mucous, and salivary glands, the gall-bladder, the bladder for urine—in short, every organ or part which is either secreting in itself or acting as a reservoir for the secretions of other parts, must furnish an illustration of the truth of our remark.

If the testicle continues so sound as to secrete, we question, in the first place, whether any operation is required, (except in particular cases,) and, in the next, we maintain, that the effect of the excision of the excretory tube must inevitably be the accumulation and distension in the testis or epididymis, a condition, we should think, that would rather bring on a disease than relieve it. If the testicle is disorganized and so utterly *hors de combat*, as not to secrete any longer, the accumulation of course will be prevented, but *then* the vas deferens has lost the connexion of function with the testicle; it has ceased to be an excretory tube, for the palpable reason that there is nothing to excrete, and the expectation of removing a disease in the one by excision of the other, would be as chimerical a notion and futile a whim as was ever engendered in a crazed imagination.

We are told that the operation has already been successful in Germany, but we do not believe it. The operation has been done, and the patient very probably got well; but when we bear in memory the absolute ignorance of the diagnosis of diseases of the testicle which prevails upon the Continent, and too often in this country also, we receive every statement of the kind with even more than a granum salis!

It appears that Mr. Lambert, the reporter for the Borough that *was*,* has instituted a series of experiments on the subject, "which have attracted much attention," though, in our ignorance, we had never even heard of them. They are, no doubt, conclusive on the point, and will fell to the ground "with the powers of an Afrite," the arguments we have brought forward against the operation. However, we submit with resignation to the castigation we shall certainly receive, for presuming to array common sense against the important and attracting experiments of so clever a person as the Reporter for the Borough!

* *Fuit* Ilium.
